# Targeting Poly(ADP)ribose polymerase in BCR/ABL1-positive cells

**DOI:** 10.1038/s41598-023-33852-2

**Published:** 2023-05-10

**Authors:** Haruka Hiroki, Yuko Ishii, Jinhua Piao, Yui Namikawa, Mitsuko Masutani, Hiroaki Honda, Koshi Akahane, Takeshi Inukai, Tomohiro Morio, Masatoshi Takagi

**Affiliations:** 1 Department of Pediatrics and Developmental Biology, Tokyo Medical and Dental University (TMDU), Yushima 1-5-45, Bunkyo-Ku, Tokyo, 113-8519 Japan; 2 Department of Molecular and Genomic Biomedicine, Center for Bioinformatics and Molecular Medicine, Nagasaki University Graduate School of Biomedical Sciences, 852-8523 Nagasaki, Japan; 3 Division of Cellular Signaling, National Cancer Center Research Institute, Tokyo, Japan; 4 Field of Human Disease Models, Major in Advanced Life Sciences and Medicine, Institute of Laboratory Animals, Tokyo Women’s Medical University, Tokyo, Japan; 5 Department of Pediatrics, School of Medicine, University of Yamanashi, Yamanashi, Japan

**Keywords:** Acute lymphocytic leukaemia, Chronic myeloid leukaemia, Apoptosis, Leukaemia

## Abstract

BCR/ABL1 causes dysregulated cell proliferation and is responsible for chronic myelogenous leukemia (CML) and Philadelphia chromosome-positive acute lymphoblastic leukemia (Ph1-ALL). In addition to the deregulatory effects of its kinase activity on cell proliferation, BCR/ABL1 induces genomic instability by downregulating BRCA1. PARP inhibitors (PARPi) effectively induce cell death in BRCA-defective cells. Therefore, PARPi are expected to inhibit growth of CML and Ph1-ALL cells showing downregulated expression of BRCA1. Here, we show that PARPi effectively induced cell death in BCR/ABL1 positive cells and suppressed colony forming activity. Prevention of BCR/ABL1-mediated leukemogenesis by PARP inhibition was tested in two in vivo models: wild-type mice that had undergone hematopoietic cell transplantation with BCR/ABL1-transduced cells, and a genetic model constructed by crossing *Parp1* knockout mice with BCR/ABL1 transgenic mice. The results showed that a PARPi, olaparib, attenuates BCR/ABL1-mediated leukemogenesis. One possible mechanism underlying PARPi-dependent inhibition of leukemogenesis is increased interferon signaling via activation of the cGAS/STING pathway. This is compatible with the use of interferon as a first-line therapy for CML. Because tyrosine kinase inhibitor (TKI) monotherapy does not completely eradicate leukemic cells in all patients, combined use of PARPi and a TKI is an attractive option that may eradicate CML stem cells.

## Introduction

BCR/ABL1 plays a key role in development of chronic myelogenous leukemia (CML) and in some cases of Philadelphia chromosome-positive acute lymphoblastic leukemia (Ph1-ALL). Multiple signaling pathways, mainly those associated with cellular proliferation, are activated by BCR/ABL1^1^. Previously, we showed that the DNA damage response pathway in the bone marrow of chronic-phase CML patients is activated, possibly due to enforced proliferation signals driven by BCR/ABL1; disruption of the DNA damage response machinery increased susceptibility of CML patients to blast crisis^2^. Other reports demonstrate induction of genomic instability in BCR/ABL1-expressing cells upon attenuation of BRCA1^3,4^. BCR/ABL1-mediated downregulation of BRCA1 is induced by translational repression and increased protein degradation^5,6^.

BRCA1 plays a critical role in homologous recombination repair (HRR), and innate defects in BRCA1 cause hereditary breast and ovarian cancer (HBOC). Current therapeutic strategies for HBOC include PARP inhibitors (PARPi) such as olaparib. PARPi increase the number of unrepaired single-strand breaks (SSB) caused by attenuated base excision repair. SSBs are converted to single-ended double-strand breaks (DSB) during replication and which are repaired by BRCA1-competent HRR mechanisms. Thus, PARPi are synthetic lethal to HRR-defective cells^7^. In addition to BRCA1, defects in molecules involved in HRR also increase sensitization to PARPi^8^. These observations suggest that BCR/ABL1-mediated downregulation of BRCA1 makes cells susceptible to PARPi.

## Results

### Effect of olaparib on leukemia cells

**Figure 1:**
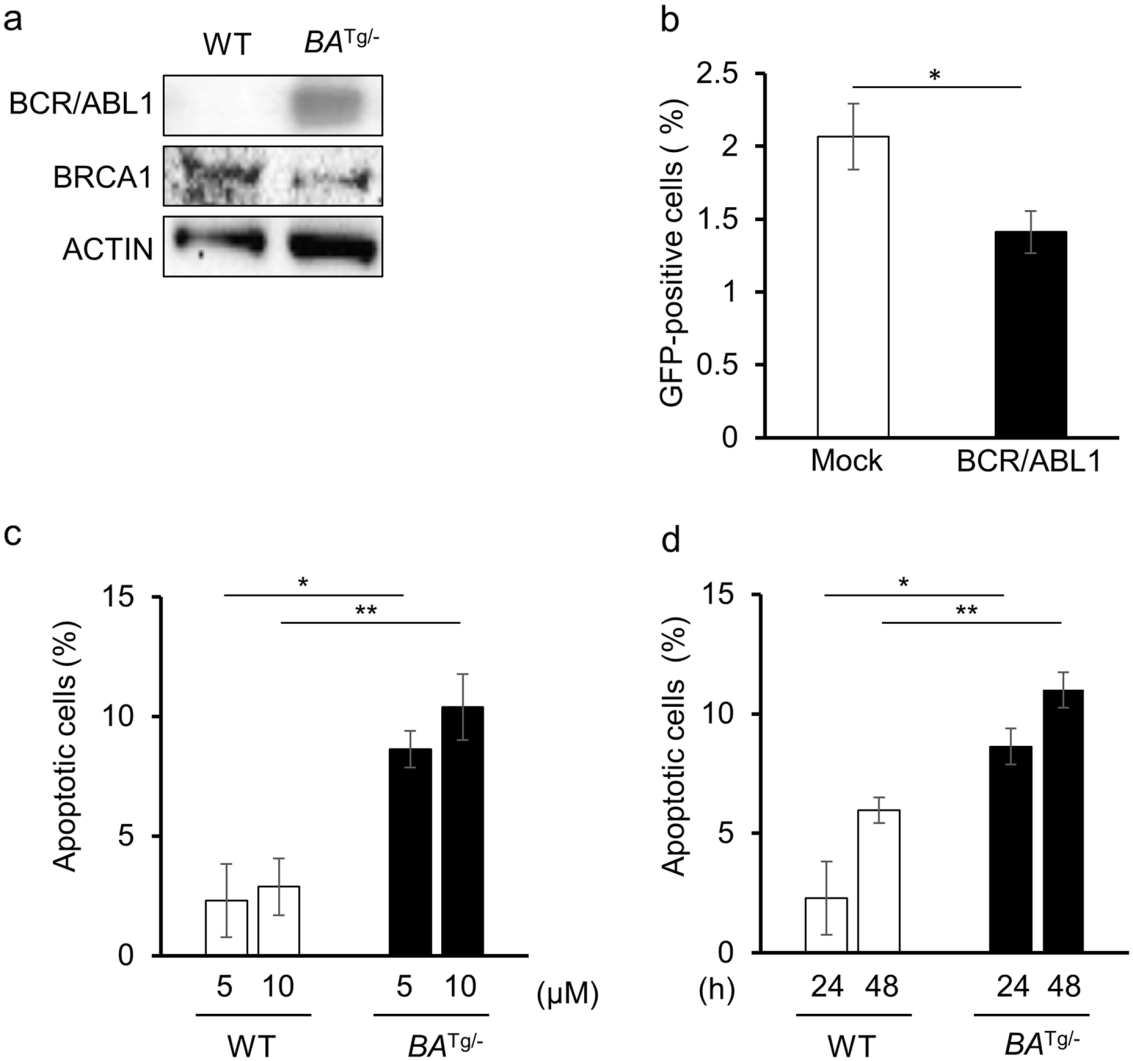
Sensitivity of BCR/ABL1-expressing leukemic cells to homologous recombination repair defects (HRD) and PARPi. (**a**) Immunoblot showing expression of BRCA1 in hematopoietic cells from BCR/ABL1-transgenic mice and wild-type (WT) mice. (**b**) BCR/ABL1 suppresses HRR activity. HRR activity was assessed in mock- or BCR/ABL1-transduced MRC5SV cells harboring a single integrated copy of DR-GFP. GFP expression was measured by flow cytometry at 48 h post-transfection of I-SceI. The percentages of GFP-positive cells are shown in the bar graph. *p = 0.03. (**c**) (**d**) Percentage of apoptotic hematopoietic mononuclear cells derived from WT and BCR/ABL1 Tg mice. Cells were treated for 24 h with the indicated concentrations of olaparib (*p = 0.02 and **p = 0.01) (**c**), or with 5 μM of olaparib, for the indicated times (**d**) (*p = 0.02, **p = 0.01). The percentage of induced apoptotic cells was obtained by subtracting the percentage of untreated cells. The data in the bar graph represent the mean ± standard deviation (SD) of three independent experiments.

Expression of BRCA1 by bone marrow-derived cells from BCR/ABL1 transgenic (Tg) mice was lower than that by cells from wild-type (WT) mice (Fig. 1a). BRCA1 is a key molecule involved in the HRR pathway. Therefore, we examined the effect of BCR/ABL1 expression on HRR activity. As expected, HRR activity was downregulated upon expression of BCR/ABL1 (Fig. 1b). These results suggest that BCR/ABL1-expressing cells exhibit homologous recombination defects (HRD). Next, bone marrow-derived mononuclear cells (MNC) from WT and BCR/ABL1 Tg mice were exposed to the PARPi olaparib in vitro, and cell death was analyzed by Annexin V/propidium iodide staining. Olaparib induced cell death in both WT and BCR/ABL1 Tg mouse-derived MNCs in a dose- and time-dependent manner. BCR/ABL1 Tg mouse-derived MNCs were more sensitive to olaparib than those from WT mice (Fig. 1c,d).

### Olaparib prevents transformation activity by BCR/ABL1

It would be interesting to ascertain whether olaparib prevents transformation, or whether PARP is required for transformation by BCR/ABL1. Therefore, we examined BCR/ABL1-mediated transformation activity in Rat-1 cells^12^. Rat-1 cells were either mock-infected or infected with BCR/ABL1 (Supplemental Fig. 1a), and colony formation activity was monitored in the presence or absence of olaparib. Treatment with olaparib reduced the colony transformation activity of BCR/ABL1 (Supplemental Fig. 1b,c).

### Olaparib reduces the potential of BCR/ABL1-expressing cells to repopulate HSCs

**Figure 2:**
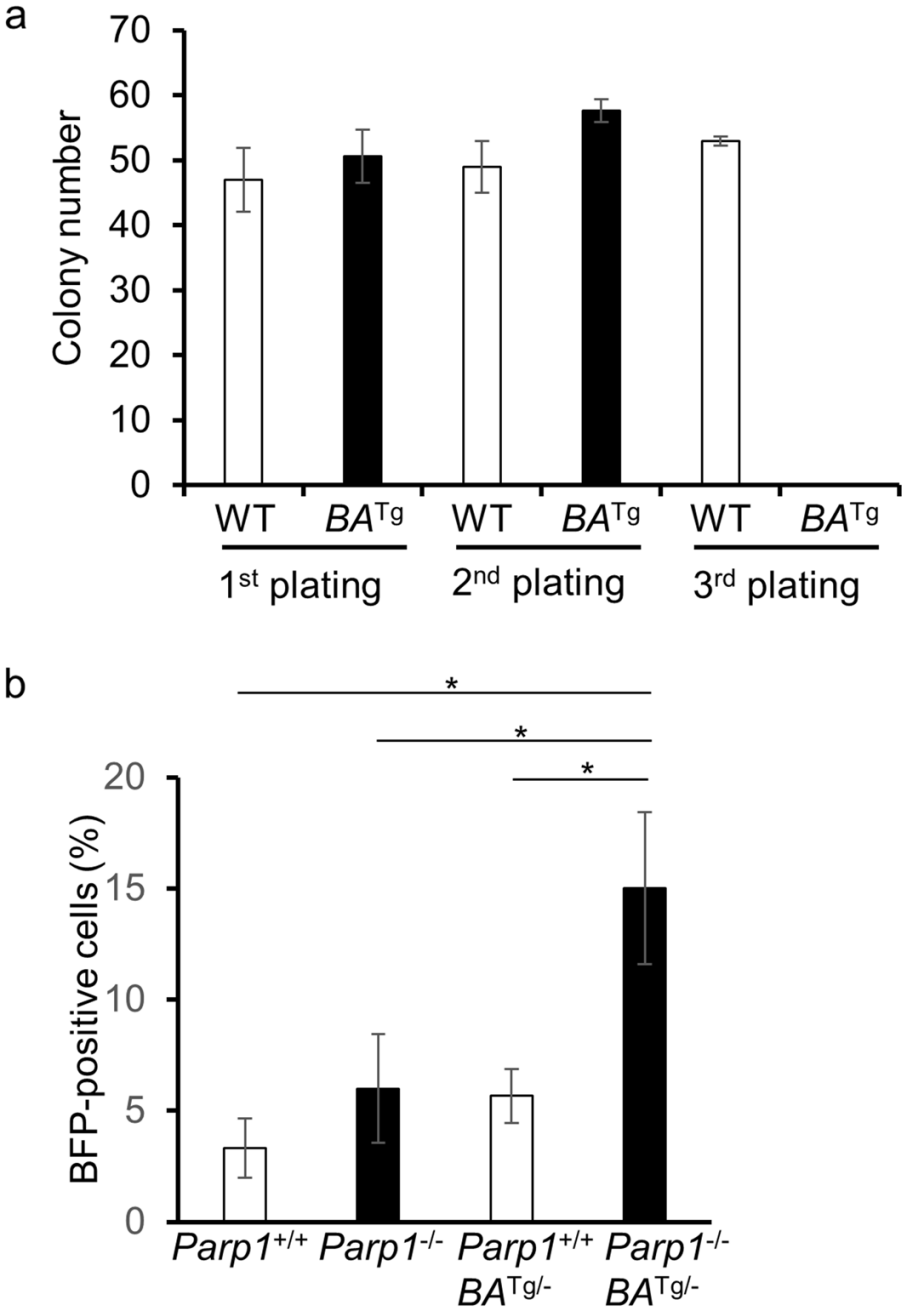
Olaparib reduces HSC potential in BCR/ABL1-expressing cells. (**a**) The colony-forming activity of HSCs from wild-type (WT) or BCR/ABL1 transgenic mice (*BA*^*Tg*^) was analyzed in serial replating experiments. Cells were maintained on agar containing 5 μM olaparib. Colony numbers on each plate are shown in the bar graph. The average colony number from three independent experiments (mean ± SD) is shown. (**b**) BFB formation was assessed in bone marrow hematopoietic stem cells (Lin^-^c-kit^+^Sca1^+^CD105^+^) obtained from *Parp*^+/+^, *Parp1*^-/-^, *Parp1*^+/+^*BA*^Tg/-^, and *Parp1*^-/-^*BA*^Tg/-^ mice. The percentages of BFB-positive cells are shown in the bar graph (mean ± SD of three independent experiments). *p = 0.05.

Next, we performed colony assays to examine the repopulation activity of HSCs in order to analyze the effect of PARP inhibition. The colony-forming activity of HSCs from WT and BCR/ABL1 Tg mice was examined sequentially after replating under continuous olaparib exposure. Intriguingly, colony-forming activity by BCR/ABL1-expressing HSCs was abolished after the third replating, whereas wild-type HSCs retained this activity (Fig. 2a).

We hypothesized that genomic instability reduces the HSC-repopulating potential of BCR/ABL1-expressing cells. Usually, HSCs arrest at G0 and only enter the cell cycle if they are stimulated. Also, CML progenitors demonstrate increased susceptibility to repeated cycles of chromosome damage, repair, and damage via a breakage-fusion-bridge (BFB) mechanism^13^. Therefore, we examined BFB generation under conditions of cytokine stimulation. Even though cells were stimulated with cytokines, the number of wild-type cells was not different from that of BCR/ABL1-expressing cells. As expected, as cells progressed through the cell cycle, the number of BFBs in the *Parp1*^-/-^ and *Parp1*^+/+^*BA*^Tg/−^ HSC population increased moderately, whereas the number in the *Parp1*^−/−^*BA*^Tg/−^ HSC population was markedly higher than that in the *Parp1*^+/+^, *Parp1*^−/−^, and *Parp1*^+/+^*BA*^Tg/−^ HSC populations (Fig. 2b and Supplemental Fig. 1d).

Next, to gain insight into how olaparib affects stem cell maintenance, we compared differentially expressed genes between DMSO-treated and olaparib-treated cells using RNA sequencing-based transcriptome analysis. When we focused on highly expressed genes in olaparib -treated cells, we identified the genes associated with the TP53 signaling pathway (Table S1 and Supplemental Fig. 2a). Conversely, a focus on downregulated genes in olaparib-treated cells identified genes involved in oxphosphorylation (OXPHOS) (Table S1 and Supplemental Fig. 2b).

### Activation of the cGAS/STING pathway in BCR/ABL1-expressing cells

**Figure 3:**
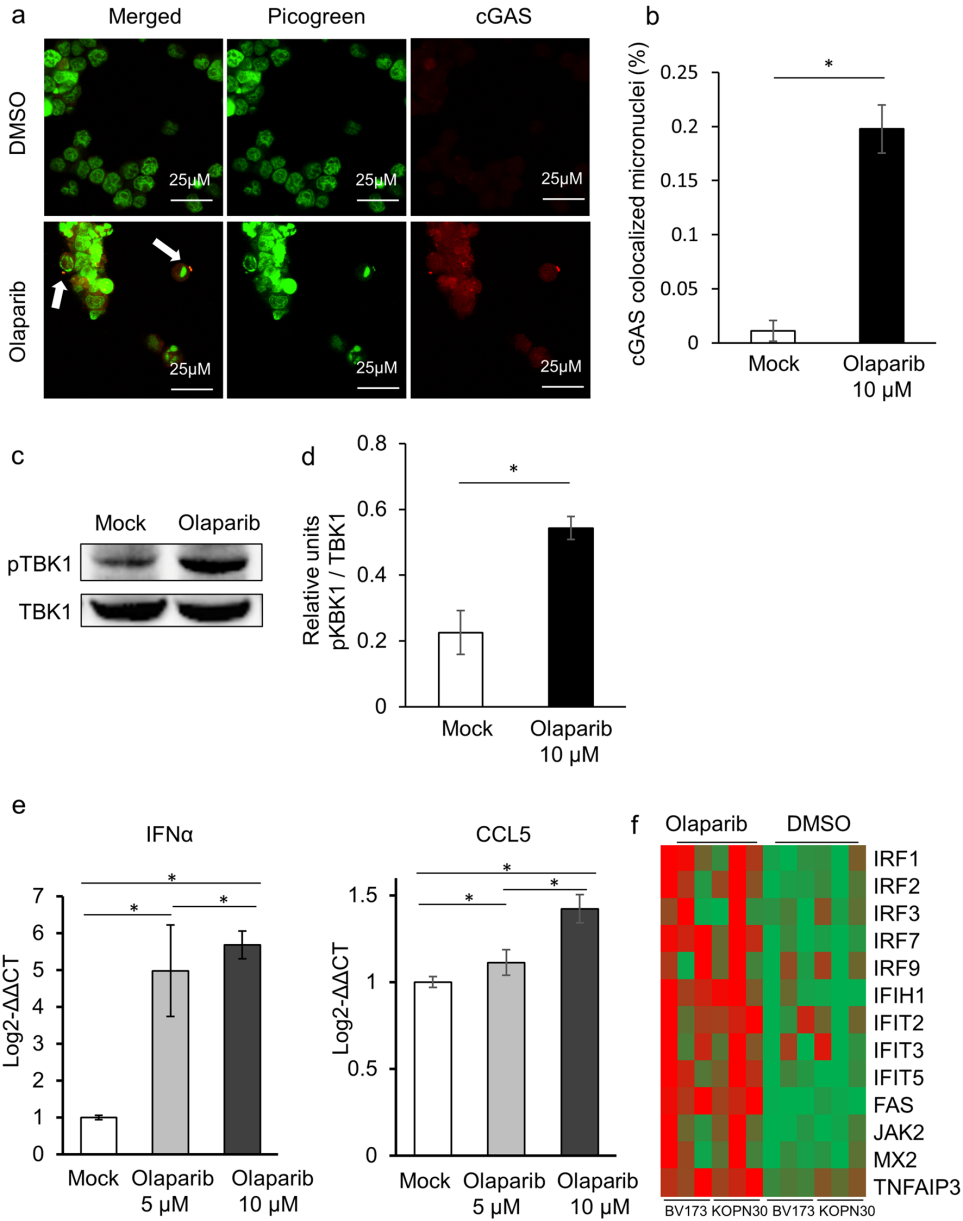
Olaparib activates the cGAS/STING pathway. (**a**), (**b**) Olaparib induces cGAS-bound micronuclei. (**a**) Representative immunofluorescence images of cGAS-colocalized micronuclei. BV173 cells were incubated for 24 h with DMSO or 10 μM olaparib and then subjected to immunofluorescence with pico488 (green) and an anti-cGAS antibody (red). White arrows show cGAS-colocalized micronuclei. (**b**) The percentage of cells with cGAS-colocalized micronuclei. The data in the bar graph represent the mean ± SD of three independent experiments. *p = 0.05. (**c**), (**d**) Activation of the cGAS/STING pathway was monitored by measuring TBK1 phosphorylation. (**c**) Immunoblot showing expression of pTBK1 and aTBK1 in BV173 cells treated with DMSO or 10 μM olaparib for 18 h before immunoprecipitation and western blotting. (**d**) pTBK1/TBK1, expressed as relative units. The data in the bar graph represent the mean ± SD of three independent experiments. *p = 0.05. € Expression of IFN-α and CCL5 was measured by RT-qPCR. BV173 cells were treated with 5 or 10 μM olaparib for 12 h. *p = 0.05. The data in the bar graph represent the mean ± SD of three independent experiments. (**f**) Expression of interferon-induced genes in DMSO-treated or olaparib-treated BCR/ABL1-positive cell lines (BV173 and KOPN30, n = 3, respectively). Cells were treated for 12 h with 10 μM olaparib or DMSO. Gene expression is shown as a heatmap.

PARPi have a broad range of biological effects^21^ that may have caused the observed reduction in survival of BCR/ABL1-positive cells. The cGAS/STING pathway, which is responsible for de novo synthesis of antiviral type I interferons (IFNs) and their related gene products, is triggered by cytosolic DNA to induce antitumor immune responses^22^. Accumulation of DNA damage following PARP inhibition leads to leakage of damaged double-stranded DNA into the cell cytoplasm, which activates innate immune signaling through the cGAS–STING pathway, leading to increased expression and release of type I IFN^17,23,24^. IFN was once the standard frontline treatment for CML because its pleiotropic mechanism of action includes immune activation and specific targeting of CML stem cells^25^. Therefore, based on the hypothesis that cGAS/STING pathway-mediated activation of the IFN machinery exerts cytotoxic effects on CML LSC, we investigated the effect of olaparib on activation of the cGAS/STING pathway. As expected, olaparib induced cGAS-bound micronuclei (Fig. 3a,b) and TBK1 phosphorylation, which is crucial for STNG activation (Fig. 3c,d). Furthermore, we observed increased expression of IFN-α and CCL5 mRNA (Fig. 3e, and Supplemental Fig. 3a,b). RNA sequencing also revealed upregulation of IFN-responsive genes (Fig. 3f).

### Olaparib inhibits BCR/ABL1-dependent leukemia in vivo

**Figure 4:**
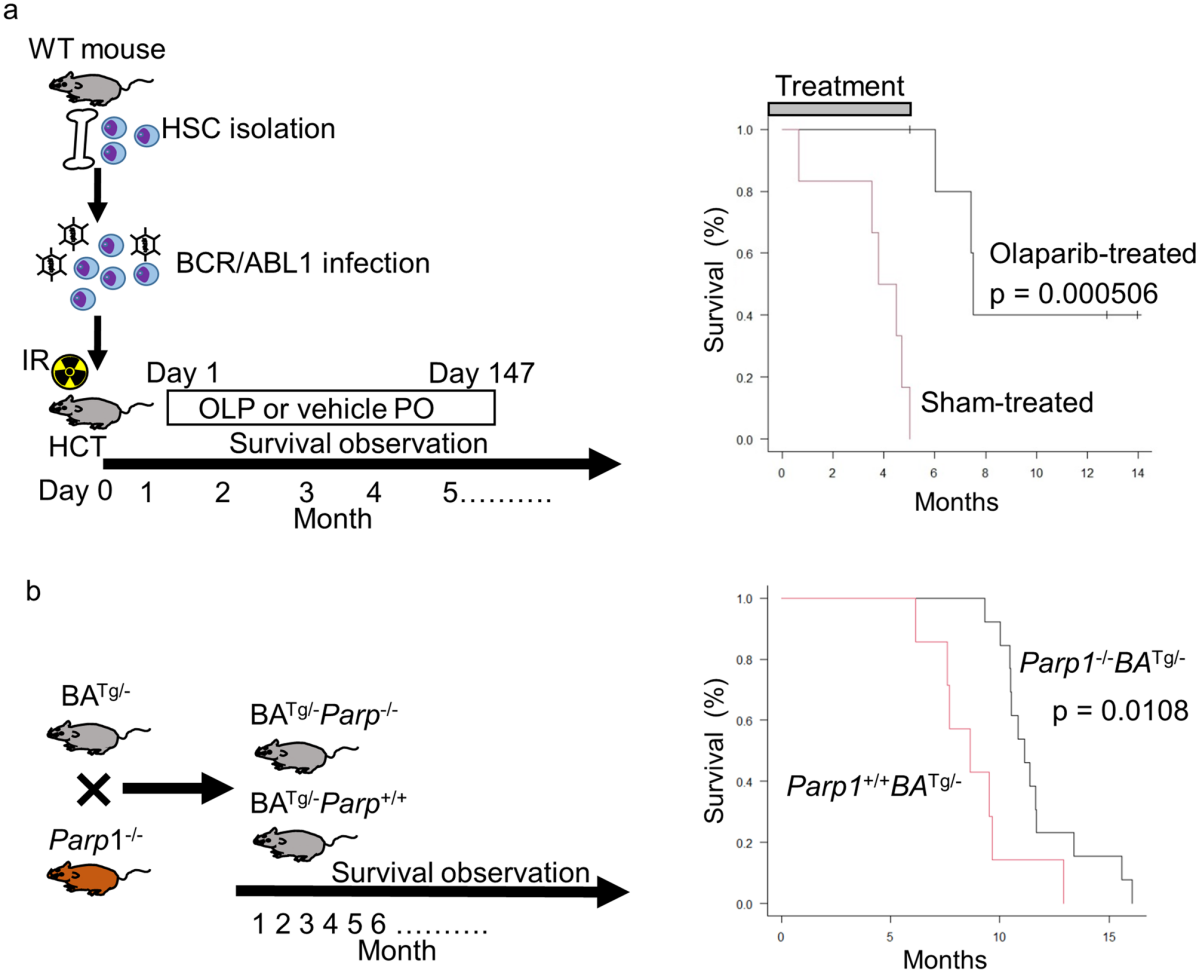
Survival of a BCR/ABL1-mediated leukemia mouse model after targeting PARP. (**a**) Study design, and Kaplan–Meier survival curves for mice transplanted with BCR/ABL1-expressing HSCs. Mice (n = 6 per group) received vehicle (10% 2-hydroxypropyl-β-cyclodextrin [sham]) or olaparib (100 mg/kg dissolved in vehicle) orally five days per week, starting on Day 1 post-transplantation and ending at the time of death. Survival was monitored until all vehicle-treated mice were dead. All olaparib-treated mice remained alive after all vehicle-treated mice had died. (**b**) Study design, and Kaplan–Meier survival curves for *Parp1*^+/+^*BA*^Tg/−^ or *Parp1*^−/−^*BA*^Tg/−^ mice (*Parp1*^+/+^*BA*^Tg/−^, n = 7; *Parp1*^−/−^*BA*^Tg/−^, n = 12).

Next, we evaluated the effects of PARP inhibition in BCR/ABL1-expressing cells using an in vivo model of hematopoietic cell transplantation. Mouse HSCs were infected with a BCR/ABL1-expressing retrovirus and then transplanted into lethally irradiated mice. Starting at 1 day post-transplantation, mice received an oral 100 mg/kg olaparib (five times per week) or vehicle. Death from BCR/ABL1-mediated leukemia was observed in sham-treated mice at 1 month post-transplantation. All of the mice that received vehicle died within 6 months. However, none of the olaparib-treated mice developed leukemia, and all survived for 6 months (Fig. 4a). After all sham treated mice had died, olaparib administration to the other mice was terminated, and their survival was monitored for about 12 months post-transplantation. Three out of six mice died after termination of olaparib. Overall survival was 50%. Thus, olaparib extends survival significantly (*p* = 0.0005).

### *Parp1* knockout increases survival of BCR/ABL1 transgenic mice

To further confirm the role of PARP inhibition in preventing BCR/ABL1-mediated leukemogenesis, we used a genetic approach. Instead of inhibiting PARP using an inhibitor, we crossed *Parp1* knockout mice with BCR/ABL1 Tg mice and examined leukemia development and death. As shown previously, leukemia in BCR/ABL1 Tg mice developed 6 months after birth, after which time the mice started to die^18^. Although there was no difference in white blood cell counts between *Parp1* wild-type (*Parp1*^+/+^ *BA*^Tg/−^) and *Parp1* knockout BCR/ABL1 Tg mice (*Parp1*^−/−^ *BA*^Tg/−^) (Supplemental Fig. 4), we found it interesting that leukemia development was delayed in *Parp1*^−/−^ *BA*^Tg/−^; these mice survived longer than *Parp1*^+/+^ *BA*^Tg/−^ mice (Fig. 4b).

## Discussion

Targeting BRCA1/2deficient tumors with PARPi is a standard therapeutic option for HBOC. Currently, PARPi are being developed to target not only BRCA1/2defective HBOC, but also other types of cancer that harbor HRD^26^. Therefore, the genomic instability mediated by BCR/ABL1-mediated downregulation of BRCA1 expression in CML and Ph1-ALL is an attractive candidate for targeted therapy with PARPi. Here, a murine transplantation model yielded data supporting the potential of PARPi for the treatment of BCR/ABL1-positive leukemia. This result was also supported by results obtained using genetic models constructed by crossing *Parp1* knockout mice with BCR/ABL1 transgenic mice, and by previous reports^27,28^. PARPi exert their function against multiple PARP family proteins. Among them, olaparib is a potent inhibitor of PARP1 and PARP2. Here, we assessed only *Parp1* knockout mice. Although *Parp1*^−/−^ or *Parp* 2^−/−^ knockout mice are viable, *Parp*1^−/−^*Parp*2^−/−^ double knockout is lethal^29^. This difference may be due to the supporting effects of PARP2 on PARP1. We and others showed previously that PARPi have different cytotoxic effects depending on the BCR/ABL1-positive leukemic cell line^30–32^; however, these studies did not characterize differences between the cell lines^30,33^. By contrast, PARPi exerts a cytotoxic effect against MNCs and HSCs from BCR/ABL1 Tg mice. Therefore, we hypothesized that these conflicting results can be explained by different genetic changes in different cells. Accumulation of multiple genetic alterations and disruption of cell signaling pathways play crucial roles in leukemic transformation. Non-transformed cells from BCR/ABL1 Tg mice carry relatively simple genetic alterations, i.e., those that affect only BCR/ABL1 expression, which mimics the chronic phase of CML.

The results of our in vitro repopulation colony assay suggest that PARPi attenuates HSC homeostasis in BCR/ABL1-positive cells. HSCs localize in hypoxic environments and so are not able to generate ATP using oxygen-consuming mitochondrial OXPHOS. Therefore, they use the glycolytic system predominantly. Whereas CML LSCs show higher TCA cycle flux and mitochondrial respiration than their normal HSCs counterparts, LSCs rely more on OXPHOS, in keeping with their glycolytic metabolic profile^34^. Therefore, downregulation of OXPHOS genes via PARP inhibition may contribute to eradication of BCR/ABL1-positive LSCs. LSCs accumulate high levels of reactive oxygen species (ROS) and oxidative DNA damage^35^. Thus, HSCs become exhausted. HSCs from *Atm*/*Foxo* knockout mice, or from other mouse models defective in DNA repair, exhibit premature exhaustion due to accumulation of ROS and/or DNA damage^36,37^. PARP is necessary to maintain genomic integrity in HSCs. PARP1 and PARP2 display overlapping functions, as indicated by the early lethality of double mutant embryos^29^. Farees et al. report that *Parp2*^−/−^ mice exposed to sublethal doses of irradiation exhibit bone marrow failure, which correlates with reduced long-term repopulation of irradiated *Parp2*^−/−^ HSCs under competitive conditions^38^. In addition, Li et al. reported that activation of PARP1 by salidroside protects quiescent HSCs from oxidative stress-induced cycling and self-renewal defects, both of which are abrogated by genetic ablation or pharmacologic inhibition of PARP1^39^. Expression of BCR/ABL1 augments DNA damage and/or increases ROS production in HSCs. Therefore, under conditions of PARP knockout or PARP inhibition, these cells may easily become exhausted. Another explanation is that HSCs in vivo are in a quiescent state and only proliferate when stimulated. Here, we found no significant difference in the amount of DNA damage in HSCs from *Parp1*^−/−^*BA*^Tg/−^ and *Parp1*^+/+^*BA*^Tg/−^ mice; however, we observed increased genomic instability in proliferating HSCs from *Parp1*^−/−^*BA*^Tg/−^ mice. Thus, PARP inhibition may accelerate exhaustion of replicating BCR/ABL1-positive HSCs.

Historically, IFN has been used as a first-line therapy for patients with chronic-phase CML who are not eligible for allogeneic stem cell transplantation; this was the case until introduction of the potent BCR/ABL tyrosine kinase inhibitor imatinib mesylate. IFN can activate the immune system to target and eradicate CML stem cells. A subset of HSCs is highly quiescent^40^. Thus, the effects of PARPi (i.e., creation of DSBs by inhibiting SSBs) may be limited in these cells. Activation of the cGAS/STING pathway may explain the effects of PARPi on BCR/ABL1-positive LSCs.

TKIs remain the gold standard treatment for CML and Ph1-ALL. A previous study shows that combination of a TKI with PARPi increases the antileukemic effect against BCR/ABL1-positive cells^27^. TKIs have markedly improved the outcome of patients with CML. However, only 40–60% of patients with CML that shows a deep molecular response to TKIs can safely discontinue the drugs. TKI monotherapy cannot eradicate CML stem cells from all patients completely. However, we show here that PARPi are a promising candidate treatment for leukemogenesis caused by aberrant BCR/ABL1 expression alone. Combining PARPi with a TKI is an attractive option that may eradicate CML stem cells during the chronic phase of the disease.

## Material and methods

### Cells and cell culture

KOPN30, BV173, and K562 are BCR/ABL1-positive leukemia cell lines. All leukemia cell lines, as well as Ba/F3 cells, were maintained in RPMI-1640 medium supplemented with 15% fetal bovine serum (FBS) and penicillin–streptomycin (100 U/mL) at 37 °C in an atmosphere containing 5% CO_2_. KOPN30 cells were obtained from the University of Yamanashi School of Medicine (Yamanashi, Japan). BV173 and Ba/F3 cells were obtained from DSMZ (Braunschweig, Germany). K562 cells were obtained from the JCRB cell Bank (Osaka, Japan). Rat-1 cells were obtained from RIKEN cell bank (Tsukuba, Japan). All cell lines were tested for mycoplasma contamination. Rat-1 cells and the fibroblast line MRC5SV harboring a single integrated copy of DR-GFP (DR-GFP MRC5SV) were maintained in Dulbecco’s Modified Eagle’s Medium (DMEM) supplemented with 10% FBS and penicillin–streptomycin (100 units/mL) at 37 °C in an atmosphere containing 5% CO_2_.

### DR-GFP assay

The BCR/ABL1-expressing plasmid was constructed by subcloning BCR/ABL1into the MSCV plasmid. The DR-GFP assay was performed as previously described^9^. Briefly, mock or BCR/ABL1-expressing plasmids were transiently transfected into single-copy DR-GFP-integrated MRC5SV cells using X-tremeGENE 9 (Roche, Basel, Switzerland). On the next day, cells were transfected with the I-SceI expression vector pCBAS. GFP expression was monitored by flow cytometry 48 h after transfecting cells with pCBAS.

### Cell death analyses

The percentage of apoptotic cells was measured by flow cytometry after staining with a combination of Annexin V (Abcam, Cambridge, MA) and propidium iodide^10^. Growth-inhibitory effects were assessed using a Cell Counting Kit (Dojindo, Kumamoto, Japan). A combination index, used to assess synergistic effects, was calculated using CompuSyn software^11^.

### Rat-1 cell transformation assay

A BCR/ABL1-mediated transformation assay using Rat-1 cells was performed as previously described^12^.Colony number was counted on day 21. Colony-forming activity was also measured using a CytoSelect™ 96 well Cell Transformation Assay Kit (Cell Biolabs, San Diego, CA).

### Isolation of adult long-term repopulating hematopoietic stem cells (LTR-HSCs)

HSCs (Lin^-^c-kit^+^Sca1^+^CD105^+^ cell) were harvested from 6 to 10-week-old C57BL/6 mice 4 days after intraperitoneal injection of 5-fluorouracil (5-FU; 150 mg/kg). Cells were isolated using CD105 MultiSort Kit (PE), mouse (Miltenyi Biotech, Bergisch Gladbach, Germany). Next, cells (2 × 10^5^ cells/4 mL MethoCult M3434 (Stem Cell Technology, Vancouver, Canada)/Iscove’s MDM supplemented with 2% fetal bovine serum (FBS)) were placed in a Petri dish and incubated for 12 days. Next, cells were harvested, and 2 × 10^5^ cells were reincubated under the same conditions.

### Detection of leukemic stem cells using flow cytometry

Leukemic stem cells were labeled with ALDEFLUOR reagent (STEMCELL Technologies, Vancouver, Canada). Cells were stained with allophycocyanin (APC)-conjugated anti-CD38, APC-cyanin-7 (APC-Cy-7)-conjugated anti-CD34, and Brilliant Violet421 (BV421)-conjugated anti-CD133 antibodies (all from BD Biosciences, Franklin Lakes, NJ) for 30 min at 4 °C. Cells were then washed and resuspended in AKDEFLUOR assay buffer and 7-Actinomycin D (7-AAD). Next, cells were analyzed by flow cytometry using a BD Fortessa flow cytometer (BD Biosciences).

### Breakage-fusion-bridge (BFB) formation assay

Detection of nucleoplasmic bridges was used to assess BFB frequency; the assay was optimized for mouse cells as described previously^13^. Briefly, mouse HSCs were isolated using immunomagnetic columns as described above (Miltenyi Biotech) and then cultured in αMEM supplemented with 20% FCS, 50 ng/mL mouse SCF, 50 ng/mL mouse FLT3 ligand, 50 ng/mL human IL-6, and 50 ng/mL human TPO. Next, cells were exposed to 2 Gy X-ray irradiation and cultured for 48 h, followed by addition of cytochalasin-D (0.6 μg/mL) for 24 h. Then, cells were released from cytochalasin-D treatment for 2 h and exposed to cold hypotonic (0.075 M KCl) solution. Finally, cells were fixed in Carnoy fluid, dropped onto slides, stained with DAPI, and examined using a fluorescent microscope at a magnification of × 400.

### Immunoprecipitation and western blotting

Cells were lysed with RIPA buffer [50 mM Tris–HCl, pH 8.0, 150 mM NaCl, 0.1% (v/v) sodium dodecyl sulfate, 1% (v/v) Nonidet-P40, and 0.04% (v/v) sodium deoxycholate] and immunoprecipitated with an anti-TBK1 antibody (Cell Signaling Technology, Danvers, MA). Precipitates were blotted using an antiphospho-TBK1 antibody (Cell Signaling Technology). After washing, primary antibodies were detected with horseradish peroxidase (HRP)-conjugated antirabbit or antimouse secondary antibodies and an ECL kit (GE Healthcare, Chicago, IL). Images of uncropped blots are provided in Supplemental data 1–3.

### Gene expression analysis

Libraries for RNA sequencing were prepared using the NEBNext Ultra RNA Library Prep kit from Illumina (New England BioLabs) and sequenced using an Illumina NovaSeq 6000 platform in a 100–150 bp paired-end mode. Sequence reads were aligned to GRCh37 using STAR 2.7.8a^14^. Count data were calculated using featureCounts and normalized using edgeR^15^. The results of RNA sequencing were further analyzed by gene set enrichment analysis (GSEA; version 4.1.0)^16^.

### Immunofluorescence microscopy

Cells were stained with pico488 DNA quantification regent (Lumiprobe Life ACience Solutions, Wan Chai, Hong Kong) at 37 ℃ for 2 h. After washing with PBS, cells were fixed at room temperature for 10 min in 4% formaldehyde/PBS and permeabilized at room temperature for 20 min with 0.25% Triton/PBS. Blocking was performed at room temperature for 30 min with 5% BSA and 0.1% Triton/PBS. Cells were incubated with an anti-cGAS antibody (Santa Cruz Biotechnology, Heidelberg, Germany) in blocking buffer at 4 ℃ for 1 h. After washing with PBS, cells were incubated with Goat anti-Mouse IgG (H + L) Cross-Adsorbed Secondary Antibody, Alexa Fluor 647 (Invitrogen, Massachusetts, USA). Plates were imaged using TCS SP8 (Leica, Wetzlar, Germany), and images were analyzed using ImageJ/Fiji software.

### Quantitative real-time PCR (qPCR)

Total RNA was extracted using the RNeasy Mini Kit (QIAGEN, Hilden, Germany) and reverse transcribed to cDNA using the SuperScript III First-Strand Synthesis System (Invitrogen). Next, qPCR was performed using the LightCycler 480 SYBR Green I Master (Roche). The primers used for qPCR have been reported previously^17^.

### Mice

*BCR/ABL1* heterozygous transgenic mice (designated as *BA*^Tg/−^), originally on a DBA2 × C57BL/6 background^18^, and *Parp1* heterozygous knockout mice (designated as *Parp1*^+/−^) on a 129Svj × C57BL/6 background^19^ were backcrossed for more than 15 generations to bring them close to a C57BL/6 background. Next, the two mouse strains were crossed with each other. *BCR/ABL1* heterozygous transgenic mice harboring the *Parp1* wild-type allele (designated as *Parp1*^+/+^*BA*^Tg/−^) or the *Parp1* homozygous knockout allele (designated as *Parp1*^−/−^*BA*^Tg/−^), or *Parp1* homozygous knockout mice (designated as *Parp1*^−/−^), were used for the study. All mice were bred in a specific pathogen-free unit sited in the vivarium of Tokyo Medical and Dental University. Animal care and use complied with ARRIVE guidelines and were approved by the Tokyo Medical and Dental University animal care and use committee (Protocol Number 0130266A). All methods were performed in accordance with relevant institutional guidelines and regulations.

### Hematopoietic stem cell (HSC) transplantation and transduction of BCR/ABL1

The BCR/ABL1-expressing plasmid was constructed by subcloning BCR/ABL1 into the MSCV-IRES-GFP plasmid. Plat-E cells^20^, an ecotropic packaging cell line, were transfected with MSCV-BCR/ABL1-IRES-GFP using polyethyleneimine. Supernatants containing high titers of retrovirus were collected at 48 and 72 h and concentrated using a Retro-X Concentrator (TAKARA-Clontech, Ohtsu, Japan). LTR-HSCs were cultured overnight in αMEM supplemented with 20% FBS plus 50 ng/mL each of mouse stem cell factor (SCF), human IL-6, human FLT3 ligand, and human thrombopoietin (TPO). On Day 2, cells were placed in 24-well dishes coated with RetroNectin (TAKARA-Clontech, Shiga, Japan) and infected with concentrated retrovirus particles. At 60 h postinfection, retrovirus-infected LTR-HSCs were transplanted into mice that had received (6 h earlier) myeloablative conditioning with 9.5 Gy total body irradiation. Mice were allowed access (ad libitum) to water containing 1 mg/mL neomycin trisulfate salt hydrate and 100 U/mL polymyxin B sulfate salt.

### Statistical analysis

*P*-values for the DR-GFP, apoptosis, cell survival, transformation, leukemic stem cell detection, and qPCR assays were calculated using a t test. Survival curves were constructed using the Kaplan–Meier method and analyzed using the log-rank test. All statistical tests were two-sided, and a *p*-value of < 0.05 was considered significant.

## Supplementary Information

Supplementary Information 1.

Supplementary Information 2.

Supplementary Information 3.

## Data Availability

The RNA-sequencing data sets are available at the DDBJ Sequence Read Archive (DRA) under accession number DRA013034.

## References

[1] Cilloni D, Saglio G (2012). Molecular pathways: BCR-ABL, Clinical cancer research: An official journal of the American Association for. Can. Res..

[2] Takagi M, Sato M, Piao J, Miyamoto S, Isoda T, Kitagawa M, Honda H, Mizutani S (2013). ATM-dependent DNA damage-response pathway as a determinant in chronic myelogenous leukemia. DNA Repair.

[3] Skorski T (2007). Genomic instability: The cause and effect of BCR/ABL tyrosine kinase. Curr. Hematol. Malig. Rep..

[4] Deutsch E, Jarrousse S, Buet D, Dugray A, Bonnet ML, Vozenin-Brotons MC, Guilhot F, Turhan AG, Feunteun J, Bourhis J (2003). Down-regulation of BRCA1 in BCR-ABL-expressing hematopoietic cells. Blood.

[5] Podszywalow-Bartnicka P, Wolczyk M, Kusio-Kobialka M, Wolanin K, Skowronek K, Nieborowska-Skorska M, Dasgupta Y, Skorski T, Piwocka K (2014). Downregulation of BRCA1 protein in BCR-ABL1 leukemia cells depends on stress-triggered TIAR-mediated suppression of translation. Cell Cycle.

[6] Dkhissi F, Aggoune D, Pontis J, Sorel N, Piccirilli N, LeCorf A, Guilhot F, Chomel JC, Ait-Si-Ali S, Turhan AG (2015). The downregulation of BAP1 expression by BCR-ABL reduces the stability of BRCA1 in chronic myeloid leukemia. Exp Hematol.

[7] Farmer H, McCabe N, Lord CJ, Tutt AN, Johnson DA, Richardson TB, Santarosa M, Dillon KJ, Hickson I, Knights C, Martin NM, Jackson SP, Smith GC, Ashworth A (2005). Targeting the DNA repair defect in BRCA mutant cells as a therapeutic strategy. Nature.

[8] Takagi M, Yoshida M, Nemoto Y, Tamaichi H, Tsuchida R, Seki M, Uryu K, Nishii R, Miyamoto S, Saito M, Hanada R, Kaneko H, Miyano S, Kataoka K, Yoshida K, Ohira M, Hayashi Y, Nakagawara A, Ogawa S, Mizutani S, Takita J (2017). Loss of DNA damage response in neuroblastoma and utility of a PARP inhibitor. J Natl Cancer Inst.

[9] Pierce AJ, Johnson RD, Thompson LH, Jasin M (1999). XRCC3 promotes homology-directed repair of DNA damage in mammalian cells. Genes Dev..

[10] Vermes I, Haanen C, Steffens-Nakken H, Reutelingsperger C (1995). A novel assay for apoptosis. Flow cytometric detection of phosphatidylserine expression on early apoptotic cells using fluorescein labelled Annexin V. J. Immunol. Methods.

[11] Chou TC (2006). Theoretical basis, experimental design, and computerized simulation of synergism and antagonism in drug combination studies. Pharmacol. Rev..

[12] Lugo TG, Pendergast AM, Muller AJ, Witte ON (1990). Tyrosine kinase activity and transformation potency of bcr-abl oncogene products. Science.

[13] Chakraborty S, Stark JM, Sun CL, Modi H, Chen W, O'Connor TR, Forman SJ, Bhatia S, Bhatia R (2012). Chronic myelogenous leukemia stem and progenitor cells demonstrate chromosomal instability related to repeated breakage-fusion-bridge cycles mediated by increased nonhomologous end joining. Blood.

[14] Dobin A, Davis CA, Schlesinger F, Drenkow J, Zaleski C, Jha S, Batut P, Chaisson M, Gingeras TR (2013). STAR: Ultrafast universal RNA-seq aligner. Bioinformatics.

[15] Robinson MD, McCarthy DJ, Smyth GK (2010). edgeR: A Bioconductor package for differential expression analysis of digital gene expression data. Bioinformatics.

[16] Subramanian A, Tamayo P, Mootha VK, Mukherjee S, Ebert BL, Gillette MA, Paulovich A, Pomeroy SL, Golub TR, Lander ES, Mesirov JP (2005). Gene set enrichment analysis: A knowledge-based approach for interpreting genome-wide expression profiles. Proc. Natl. Acad. Sci. U. S. A..

[17] Pantelidou C, Sonzogni O, De Oliveria Taveira M, Mehta AK, Kothari A, Wang D, Visal T, Li MK, Pinto J, Castrillon JA, Cheney EM, Bouwman P, Jonkers J, Rottenberg S, Guerriero JL, Wulf GM, Shapiro GI (2019). PARP inhibitor efficacy depends on CD8(+) T-cell recruitment via intratumoral STING pathway activation in BRCA-deficient models of triple-negative breast cancer. Cancer Discov..

[18] Honda H, Oda H, Suzuki T, Takahashi T, Witte ON, Ozawa K, Ishikawa T, Yazaki Y, Hirai H (1998). Development of acute lymphoblastic leukemia and myeloproliferative disorder in transgenic mice expressing p210bcr/abl: A novel transgenic model for human Ph1-positive leukemias. Blood.

[19] Masutani M, Suzuki H, Kamada N, Watanabe M, Ueda O, Nozaki T, Jishage K, Watanabe T, Sugimoto T, Nakagama H, Ochiya T, Sugimura T (1999). Poly(ADP-ribose) polymerase gene disruption conferred mice resistant to streptozotocin-induced diabetes. Proc. Natl. Acad. Sci. U. S. A..

[20] Morita S, Kojima T, Kitamura T (2000). Plat-E: An efficient and stable system for transient packaging of retroviruses. Gene Ther..

[21] Curtin NJ, Szabo C (2020). Poly(ADP-ribose) polymerase inhibition: Past, present and future. Nat. Rev. Drug Discov..

[22] Corrales L, McWhirter SM, Dubensky TW, Gajewski TF (2016). The host STING pathway at the interface of cancer and immunity. J. Clin. Invest..

[23] Ding L, Kim HJ, Wang Q, Kearns M, Jiang T, Ohlson CE, Li BB, Xie S, Liu JF, Stover EH, Howitt BE, Bronson RT, Lazo S, Roberts TM, Freeman GJ, Konstantinopoulos PA, Matulonis UA, Zhao JJ (2018). PARP inhibition elicits STING-dependent antitumor immunity in brca1-deficient ovarian cancer. Cell Rep..

[24] Shen J, Zhao W, Ju Z, Wang L, Peng Y, Labrie M, Yap TA, Mills GB, Peng G (2019). PARPi triggers the STING-dependent immune response and enhances the therapeutic efficacy of immune checkpoint blockade independent of BRCAness. Can. Res..

[25] Talpaz M, Mercer J, Hehlmann R (2015). The interferon-alpha revival in CML. Ann. Hematol..

[26] Farago AF, Yeap BY, Stanzione M, Hung YP, Heist RS, Marcoux JP, Zhong J, Rangachari D, Barbie DA, Phat S, Myers DT, Morris R, Kem M, Dubash TD, Kennedy EA, Digumarthy SR, Sequist LV, Hata AN, Maheswaran S, Haber DA, Lawrence MS, Shaw AT, Mino-Kenudson M, Dyson NJ, Drapkin BJ (2019). Combination olaparib and temozolomide in relapsed small-cell lung cancer. Cancer Discov..

[27] Nieborowska-Skorska M, Sullivan K, Dasgupta Y, Podszywalow-Bartnicka P, Hoser G, Maifrede S, Martinez E, Di Marcantonio D, Bolton-Gillespie E, Cramer-Morales K, Lee J, Li M, Slupianek A, Gritsyuk D, Cerny-Reiterer S, Seferynska I, Stoklosa T, Bullinger L, Zhao H, Gorbunova V, Piwocka K, Valent P, Civin CI, Muschen M, Dick JE, Wang JC, Bhatia S, Bhatia R, Eppert K, Minden MD, Sykes SM, Skorski T (2017). Gene expression and mutation-guided synthetic lethality eradicates proliferating and quiescent leukemia cells. J. Clin. Invest..

[28] Sullivan-Reed K, Bolton-Gillespie E, Dasgupta Y, Langer S, Siciliano M, Nieborowska-Skorska M, Hanamshet K, Belyaeva EA, Bernhardy AJ, Lee J, Moore M, Zhao H, Valent P, Matlawska-Wasowska K, Muschen M, Bhatia S, Bhatia R, Johnson N, Wasik MA, Mazin AV, Skorski T (2018). Simultaneous targeting of PARP1 and RAD52 triggers dual synthetic lethality in BRCA-deficient tumor cells. Cell Rep..

[29] Menissier de Murcia J, Ricoul M, Tartier L, Niedergang C, Huber A, Dantzer F, Schreiber V, Ame JC, Dierich A, LeMeur M, Sabatier L, Chambon P, de Murcia G (2003). Functional interaction between PARP-1 and PARP-2 in chromosome stability and embryonic development in mouse. EMBO J..

[30] Piao J, Takai S, Kamiya T, Inukai T, Sugita K, Ohyashiki K, Delia D, Masutani M, Mizutani S, Takagi M (2017). Poly (ADP-ribose) polymerase inhibitors selectively induce cytotoxicity in TCF3-HLF-positive leukemic cells. Cancer Lett..

[31] Xiao LY, Kan WM (2017). Poly ADP-ribose polymerase inhibition suppresses cisplatin toxicity in chronic myeloid leukemia cells. Anticancer Drugs.

[32] Tobin LA, Robert C, Rapoport AP, Gojo I, Baer MR, Tomkinson AE, Rassool FV (2013). Targeting abnormal DNA double-strand break repair in tyrosine kinase inhibitor-resistant chronic myeloid leukemias. Oncogene.

[33] Tamai M, Inukai T, Kojika S, Abe M, Kagami K, Harama D, Shinohara T, Watanabe A, Oshiro H, Akahane K, Goi K, Sugihara E, Nakada S, Sugita K (2018). T315I mutation of BCR-ABL1 into human Philadelphia chromosome-positive leukemia cell lines by homologous recombination using the CRISPR/Cas9 system. Sci. Rep..

[34] Kuntz EM, Baquero P, Michie AM, Dunn K, Tardito S, Holyoake TL, Helgason GV, Gottlieb E (2017). Targeting mitochondrial oxidative phosphorylation eradicates therapy-resistant chronic myeloid leukemia stem cells. Nat. Med..

[35] Bolton-Gillespie E, Schemionek M, Klein HU, Flis S, Hoser G, Lange T, Nieborowska-Skorska M, Maier J, Kerstiens L, Koptyra M, Muller MC, Modi H, Stoklosa T, Seferynska I, Bhatia R, Holyoake TL, Koschmieder S, Skorski T (2013). Genomic instability may originate from imatinib-refractory chronic myeloid leukemia stem cells. Blood.

[36] Ito K, Hirao A, Arai F, Matsuoka S, Takubo K, Hamaguchi I, Nomiyama K, Hosokawa K, Sakurada K, Nakagata N, Ikeda Y, Mak TW, Suda T (2004). Regulation of oxidative stress by ATM is required for self-renewal of haematopoietic stem cells. Nature.

[37] Rossi DJ, Bryder D, Seita J, Nussenzweig A, Hoeijmakers J, Weissman IL (2007). Deficiencies in DNA damage repair limit the function of haematopoietic stem cells with age. Nature.

[38] Farres J, Martin-Caballero J, Martinez C, Lozano JJ, Llacuna L, Ampurdanes C, Ruiz-Herguido C, Dantzer F, Schreiber V, Villunger A, Bigas A, Yelamos J (2013). Parp-2 is required to maintain hematopoiesis following sublethal gamma-irradiation in mice. Blood.

[39] Li X, Sipple J, Pang Q, Du W (2012). Salidroside stimulates DNA repair enzyme Parp-1 activity in mouse HSC maintenance. Blood.

[40] Bernitz JM, Kim HS, MacArthur B, Sieburg H, Moore K (2016). Hematopoietic stem cells count and remember self-renewal divisions. Cell.

