# Supplementary material for: Targeting Poly(ADP)ribose polymerase in BCR/ABL1-positive cells
Source: Sci Rep. 2023 May 10;13:7588. doi: 10.1038/s41598-023-33852-2 (PMC10172294; doi:10.1038/s41598-023-33852-2)
Supplement: Supplementary file 1 — Supplementary Information 1. [file 41598_2023_33852_MOESM1_ESM.pdf]

Supplemental Data 1

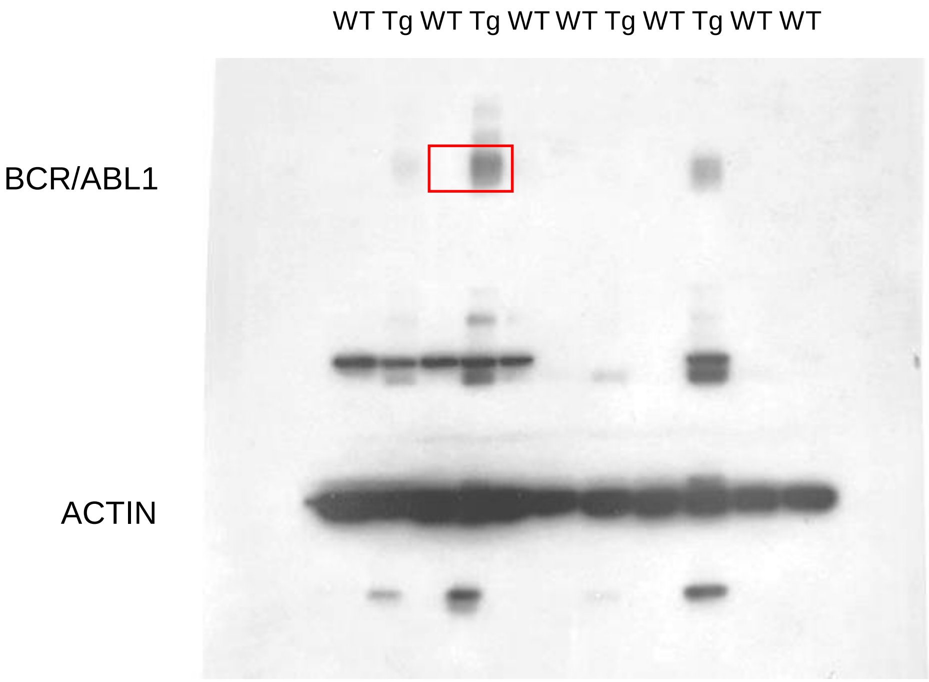

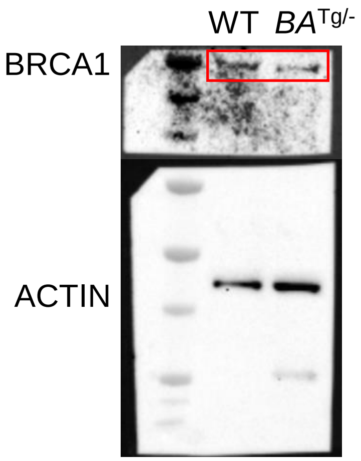

Uncropped images of the blots shown in Figure 1a. The areas enclosed by the red squares are shown in Figure 1a.

Supplemental Data 2

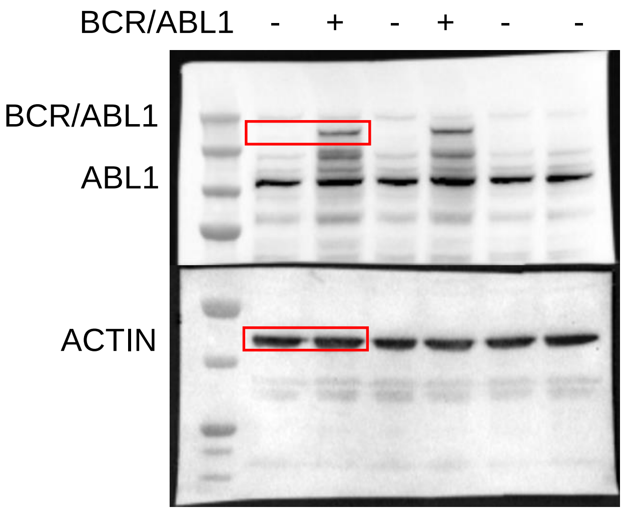

Uncropped image of the blot shown in Supplemental Figure 2a. The areas enclosed by the red squares are shown in Supplemental Fig. 2a.

Supplemental Data 3

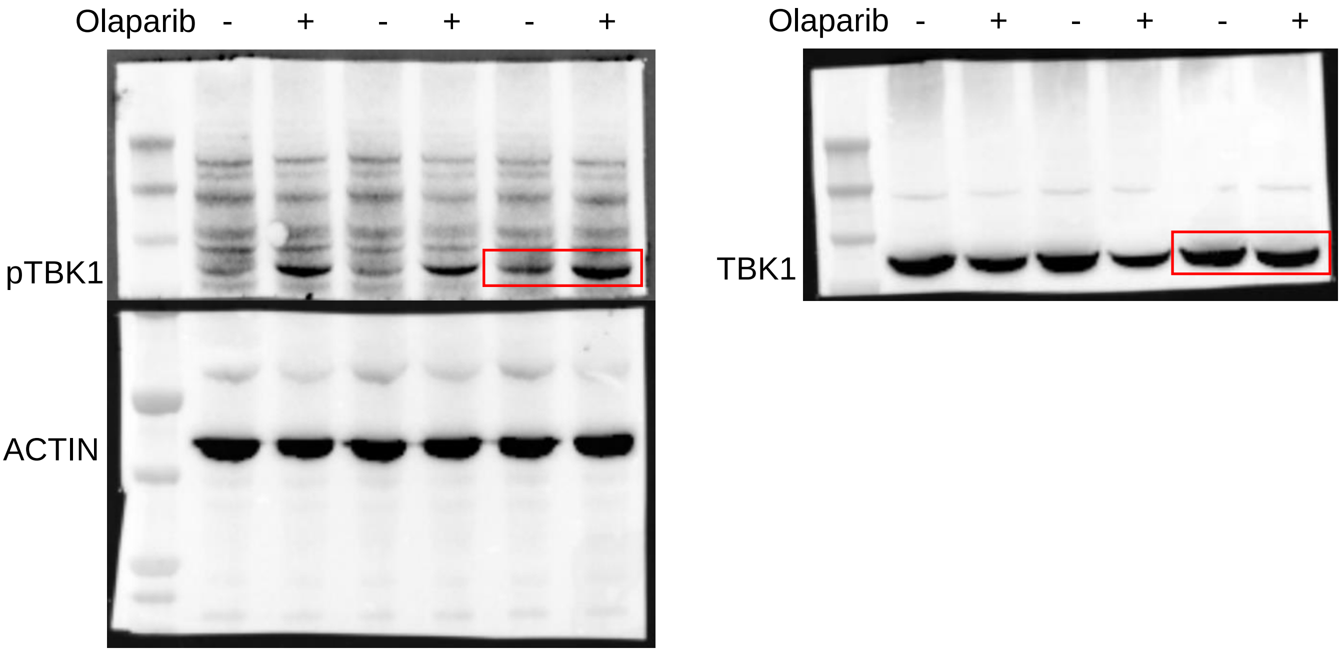

Uncropped image of the blots shown in Figure 3c. The areas enclosed by the red squares are shown in Supplemental Figure 3c.
