# Supplementary material for: Targeting Poly(ADP)ribose polymerase in BCR/ABL1-positive cells
Source: Sci Rep. 2023 May 10;13:7588. doi: 10.1038/s41598-023-33852-2 (PMC10172294; doi:10.1038/s41598-023-33852-2)
Supplement: Supplementary file 2 — Supplementary Information 2. [file 41598_2023_33852_MOESM2_ESM.pdf]

Supplemental Figure 1.

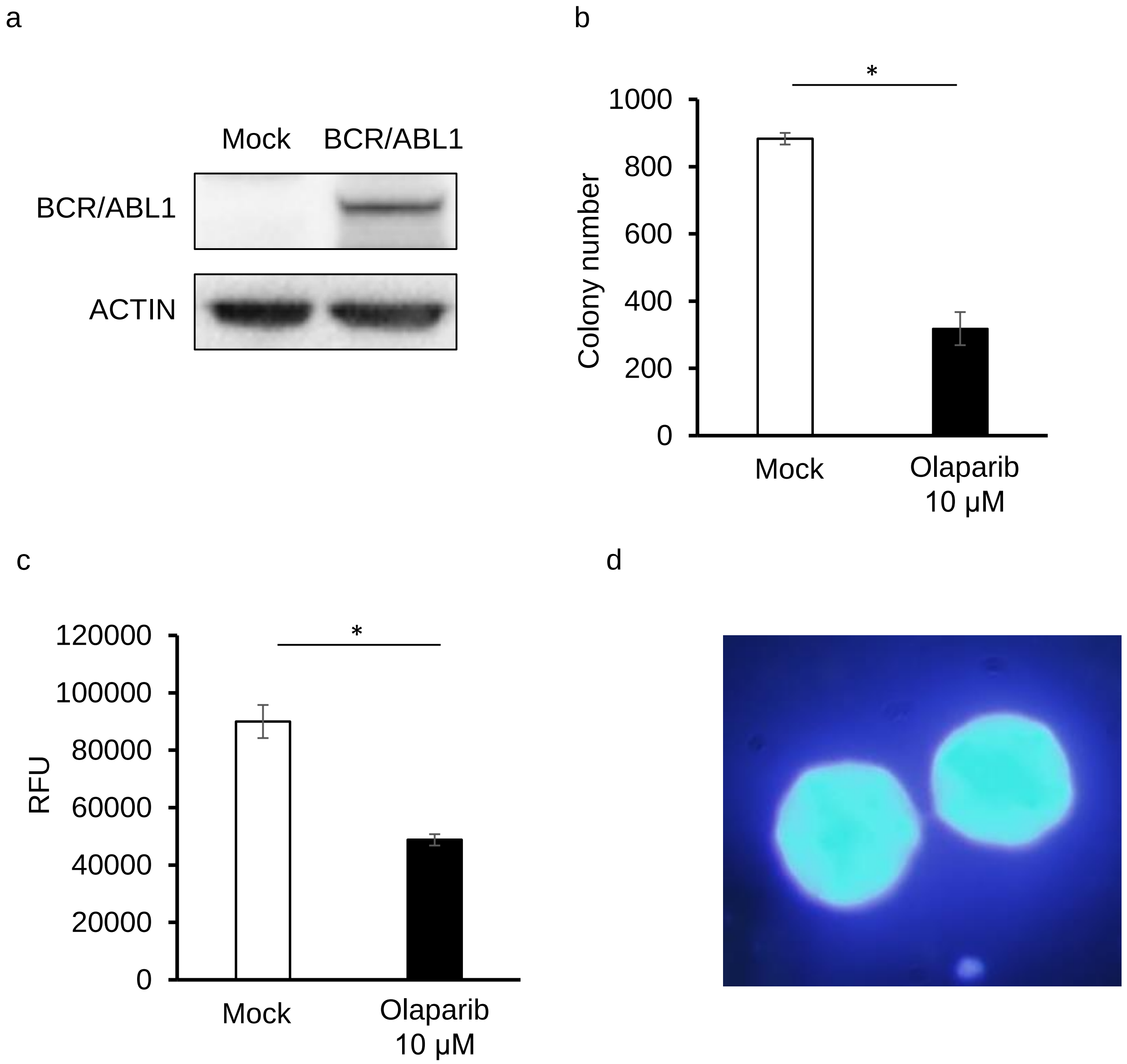

Supplemental Figure 1

Olaparib reduces BCR/ABL1-mediated transformation activity. (a) Expression of BCR/ABL1 by Rat-1 cells. The groups of blots in the panel are cropped from different parts of the same gel. (b) Transformation activity was assessed by counting colony numbers. Cells were exposed continuously to mock conditions or to 10 μM olaparib. Colonies were counted on Day 21. The average colony number counted in three independent experiments is shown (mean ± SD). * p = 0.05. (c) Transformation activity was assessed by counting colony numbers. Cells were exposed continuously to mock conditions or to 10 μM of olaparib. Colonies were counted on Day 11. Colony numbers corresponding to relative fluorescence units are shown. Data are expressed as the mean ± SD from three independent experiments. *p = 0.05. (d) A picture of BFP-positive cells.

Supplemental Figure 2.

a p53 signaling pathway

b Oxidative phosphorylation

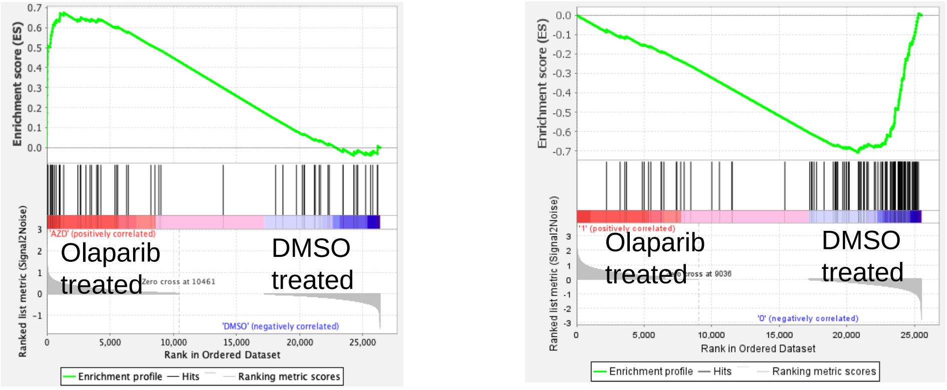

p-value: 0.0
FDR: 0.01
NES: 1.94

p-value: 0.0
FDR: 0.00
NES: -2.27815

Supplemental Figure 2

Differential expression of genes after treatment with DMSO or olaparib. (a, b) Gene expression profiling was done by GSEA. BCR/ABL1-positive cell lines (BV173 and KOPN30; each n = 3) were treated with 10 μM olaparib or DMSO. The results of GSEA are shown (d: KEGG, p53 signaling pathway; e: KEGG, oxidative phosphorylation).

Supplemental Figure 3

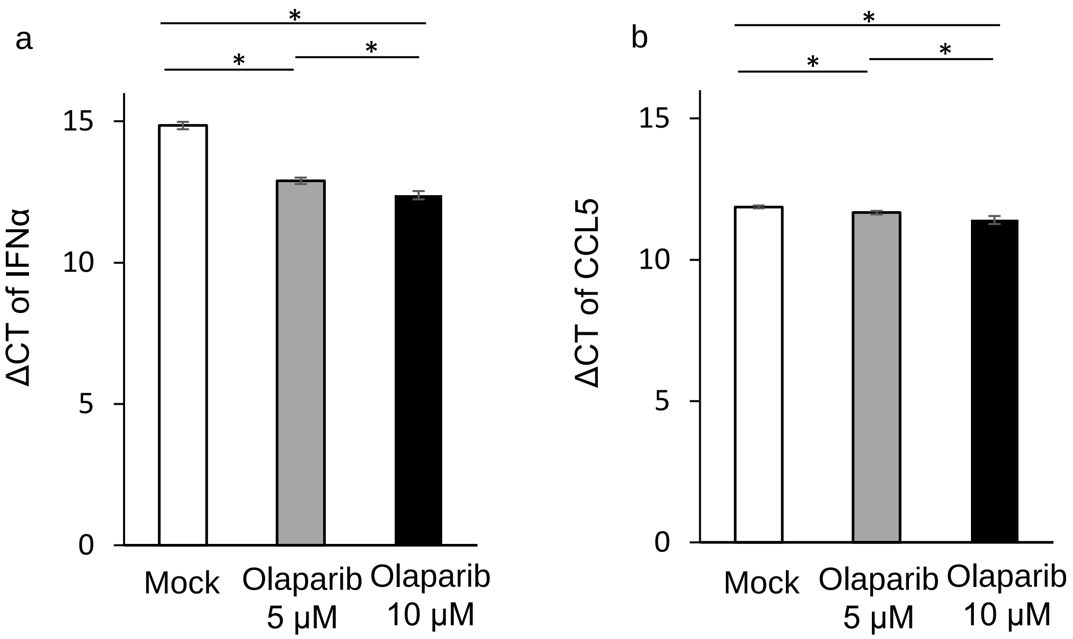

Supplemental Figure 3
Olaparib activates the cGAS/STING pathway. (a, b) ΔCT of IFNα and CCL5, as measured by RT-qPCR, is shown. BV173 cells were treated for 12 h with 5 or 10 μM olaparib. Data are expressed as the mean ± SD from three independent experiments. *p = 0.05.

Supplemental Figure 4

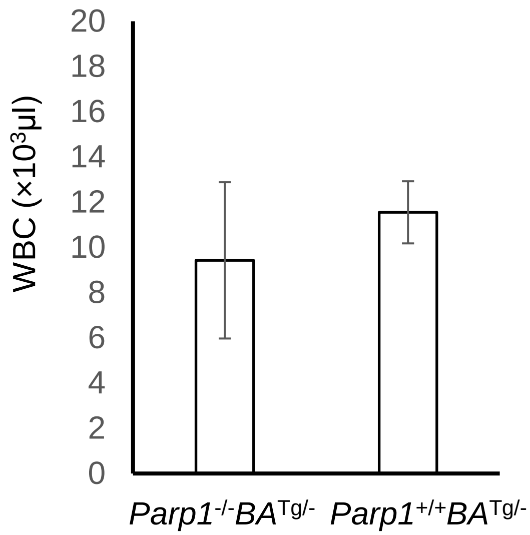

Supplemental Figure 4

Peripheral white blood cell (WBC) count in $\boldsymbol{Parp1^{-/-}BA^{Tg/-}}$ and $\boldsymbol{Parp1^{+/+}BA^{Tg/-}}$ mice. Peripheral WBC were counted at 8–11 months after birth. The bar graph shows data from four $Parp1^{-/-}BA^{Tg/-}$ and seven $Parp1^{+/+}BA^{Tg/-}$ mice (mean ± SD).
