# Supplementary material for: Targeting Poly(ADP)ribose polymerase in BCR/ABL1-positive cells
Source: Sci Rep. 2023 May 10;13:7588. doi: 10.1038/s41598-023-33852-2 (PMC10172294; doi:10.1038/s41598-023-33852-2)
Supplement: Supplementary file 3 — Supplementary Information 3. [file 41598_2023_33852_MOESM3_ESM.docx]

**Supplementary Table**

**Table S1**

**GSEA analysis of KEGG pathways upregulated by olaparib treatment of olaparib-sensitive leukemic cell lines (DMSO *vs*. olaparib)**

| NAME | SIZE | ES | NES | p value | FDR q value |
| --- | --- | --- | --- | --- | --- |
| KEGG_P53_SIGNALING_PATHWAY | 67 | 0.6740435 | 1.9628216 | 0 | 0 |

**GSEA analysis of KEGG pathways downregulated by olaparib treatment of olaparib-sensitive leukemic cell lines (DMSO *vs.* olaparib)**

| NAME | SIZE | ES | NES | p value | FDR q value |
| --- | --- | --- | --- | --- | --- |
| KEGG_OXIDATIVE_PHOSPHORYLATION | 116 | -0.70923 | -2.27815 | 0 | 0 |
| KEGG_PARKINSONS_DISEASE | 112 | -0.68545 | -2.18176 | 0 | 0 |
| KEGG_PROTEASOME | 44 | -0.79853 | -2.18107 | 0 | 0 |
| KEGG_SPLICEOSOME | 126 | -0.6267 | -2.03825 | 0 | 3.10E-04 |
| KEGG_GLYCOLYSIS_GLUCONEOGENESIS | 62 | -0.64616 | -1.91327 | 0 | 4.43E-04 |
| KEGG_CARDIAC_MUSCLE_CONTRACTION | 73 | -0.59973 | -1.80651 | 0 | 0.003481 |
| KEGG_HUNTINGTONS_DISEASE | 172 | -0.52637 | -1.78379 | 0 | 0.005065 |
| KEGG_STARCH_AND_SUCROSE_METABOLISM | 52 | -0.61413 | -1.73625 | 0 | 0.009367 |
| KEGG_GALACTOSE_METABOLISM | 26 | -0.70338 | -1.7289 | 0.002347 | 0.008816 |
| KEGG_ARGININE_AND_PROLINE_METABOLISM | 54 | -0.56173 | -1.61666 | 0 | 0.037615 |
| KEGG_FRUCTOSE_AND_MANNOSE_METABOLISM | 34 | -0.61753 | -1.61521 | 0.013274 | 0.034288 |
| KEGG_RIBOSOME | 87 | -0.52322 | -1.6075 | 0 | 0.033913 |
| KEGG_PRIMARY_IMMUNODEFICIENCY | 35 | -0.6025 | -1.59898 | 0.00978 | 0.034562 |
| KEGG_PURINE_METABOLISM | 159 | -0.47461 | -1.5658 | 0 | 0.044203 |
| KEGG_RNA_POLYMERASE | 29 | -0.61889 | -1.55207 | 0.017131 | 0.047513 |
| KEGG_PENTOSE_PHOSPHATE_PATHWAY | 27 | -0.61051 | -1.54799 | 0.024229 | 0.046271 |
| KEGG_CITRATE_CYCLE_TCA_CYCLE | 30 | -0.60084 | -1.49539 | 0.018868 | 0.070103 |
| KEGG_PYRIMIDINE_METABOLISM | 98 | -0.4726 | -1.49244 | 0.007895 | 0.06809 |
| KEGG_ALZHEIMERS_DISEASE | 157 | -0.44901 | -1.49091 | 0 | 0.065174 |
